# Primitive Action Based Combined Task and Motion Planning for the Service Robot

**DOI:** 10.3389/frobt.2022.713470

**Published:** 2022-02-10

**Authors:** Jeongmin Jeon, Hong-ryul Jung, Francisco Yumbla, Tuan Anh Luong, Hyungpil Moon

**Affiliations:** Department of Mechanical Engineering, Sungkyunkwan University, Suwon, South Korea

**Keywords:** service robots, PDDL planning, task planning, motion planning, object manipulation

## Abstract

The need for combined task and motion planning (CTAMP) in robotics is well known as robotic technologies become more mature. The goal of CTAMP is to determine a proper sequence of a robot’s actions based on symbolic and geometric reasoning. Because of the fundamental difference in symbolic and geometric reasoning, a CTAMP system often requires an interface module between the two reasoning modules. We propose a CTAMP system in which a symbolic action sequence is generated in task planning, and each action is verified geometrically in motion planning using the off-the-shelf planners and reasoners. The approach is that a set of action models is defined with PDDL in the interface module (action library) and the required information to each planner is automatically provided by the interface module. The proposed method was successfully implemented in three simulated experiments that involve manipulation tasks. According to our findings, the proposed method is effective in responding to changes in the environment and uncertainty with errors in recognition of the environment and the robot motion control.

## 1 Introduction

A service robot requires a system to manipulate objects indoors (e.g., pour drinks). This system must allow the robot to plan several actions in order, without collisions with other objects: 1) move the robot base near the object to be manipulated; 2) move the robot arm around the object; and 3) grasp the object. To solve this problem, two approaches have been studied. First, the approach of task planning aims to determine the order of actions to grasp the object (starting from the initial state of the robot). Second, the approach of motion planning aims to calculate a collision-free path to perform each action for the robot.

A classical task planner, known as the Stanford Research Institute planning solver, was developed (Pednault, 1989), and the planning domain definition language (PDDL) was standardized as the AI planning language in the International Planning Competition (Fox and Long, 2003). The task planner uses an abstract action model using a planning language. By ignoring the feasibility of the actions and considering only the causality between them, the task planner automatically generates a sequence of actions that can reach the goal state from the initial state of the task. Therefore, it is not possible to determine the actual performance of the actions through task planning. In motion planning, collision-free paths are planned: the robot must move to the desired location while considering geometric and mechanical constraints in the actual physical space. Geometric reasoning is used to verify that the robot can move along the path (Latombe, 2012). Because probabilistic motion planning methods, such as rapidly-exploring random trees (LaValle et al., 2001), have been studied, motion planning of a high-freedom manipulator is also possible. Because each manipulation task has different constraints to consider, there are various motion planners specialized for each task, such as motion planning for grasp (Fan and Tomizuka, 2019), handover (Wan et al., 2019), and pouring (Tsuchiya et al., 2019). Task planners and motion planners have been studied in different ways to solve problems for each planning purpose. The combined task and motion planning (CTAMP), which automatically determines the sequence of feasible actions, is a challenging issue for service robots.

The first method for integrating two planners includes calling the modularized motion planner whenever an action is determined by the task planner to confirm the feasibility of the action (Cambon et al., 2009; Plaku and Hager, 2010). Lagriffoul et al. (2012) used geometric backtracking to select and verify all the actions that can be performed until the robot reaches its goal state. However, calling a motion planners for every action has a disadvantage of high computational cost. Therefore, Bidot et al. (2017) suggested reducing the search space of backtracking by limiting the grasping posture or the position of placing an object.

In a different approach, a second method to integrating the two planners is to call the motion planner only for candidate actions of the task (Pandey et al., 2012; de Silva et al., 2013). This requires defining the relationship between the action model and the motion planner. Srivastava et al. (2014) used geometric parameters such as grasp postures or object positions in the action model in advance to construct the predicates of the action precondition so that the motion planning can interfere with the task planning. They did task planning first and implemented an interface layer to call the motion planner for each action. However, they take a long time to solve to relocate obstacles because they did not consider linking with additional geometric reasoning modules to make more efficient plans (Lee and Kim, 2019). Wells et al. (2019); Akbari et al. (2019) reduced the computational cost of the motion planner by calling it only if each action is verified by a geometric reasoner.

When using the CTAMP presented in the above studies to perform manipulation tasks, the robot will operate only after the results of the task plan are verified. Therefore, if the task environment changes during the calculation time, the task may fail. Moreover, there was no discussion of replanning the task if it fails due to an uncertainty error in recognition or control in scenarios with real-world robots and tasks.

In this paper, we present a CTAMP system that can be applied to various manipulation tasks and enables re-planning the task, as a way of performing motion planning on the sequence of actions obtained as a result of carrying out the task planning first. Assuming that the task requires several obstacles to be removed to grasp a target object, the action of removing the obstacle should be accompanied by such consequences as “obstacles being removed” as opposed to simply grasping or putting the object. For this reason, for the task planning part, the action of removing obstacles should be defined with more complex constraints. The action of “removing obstacles” can be seen as a compound action composed of several primitive actions (such as move the robot arm near the object, grasp it, and move the arm to another location). Therefore, in the motion planning phase, when calculating the joint trajectories for the action of removing the obstacle, several motion planners must be used to create a joint trajectory for the robot arm to grasp the obstacle and relocate it to another location. Moreover, additional geometric reasoning algorithms should be used to efficiently calculate where the obstacles should be relocated.

For that purpose, in this study, we implemented the action library to define the relationships between actions and the action-motion planner. The proposed action library informs us which compound actions consist of which primitive actions, and which motion planner can be used to verify each action’s feasibility. Using the action library, we implemented the task manager module to automatically perform the task planning for the current state and the goal state. Moreover, we implemented the behavior manager module, which manages the modularized state-of-the-art motion planners and geometric reasoners. In the proposed CTAMP system, when the sequence of primitive action was first determined by the task planner, we proposed an interference method, which automatically calls the motion planners and geometric reasoners required for the execution of each primitive action. Therefore, we do not only verify the feasibility of actions and create a plan of motions at the planning phase, but we also propose a system for re-planning. Thus, the robot can respond to task failure (caused by changes in the environment or by uncertainty errors) by performing the action whenever verification of each action is completed.

The remainder of this paper is structured as follows. Section 3 presents how the action library defines an action model using the task planning language. Section 4 describes how the task manager module automatically plans the tasks. Section 5 describes how the behavior manager calls geometric algorithms to perform primitive actions as a result of the task plan. Section 6 discusses the results of several manipulation tasks by applying the proposed system.

## 2 Proposed Task and Motion Planning System

**FIGURE 1:**
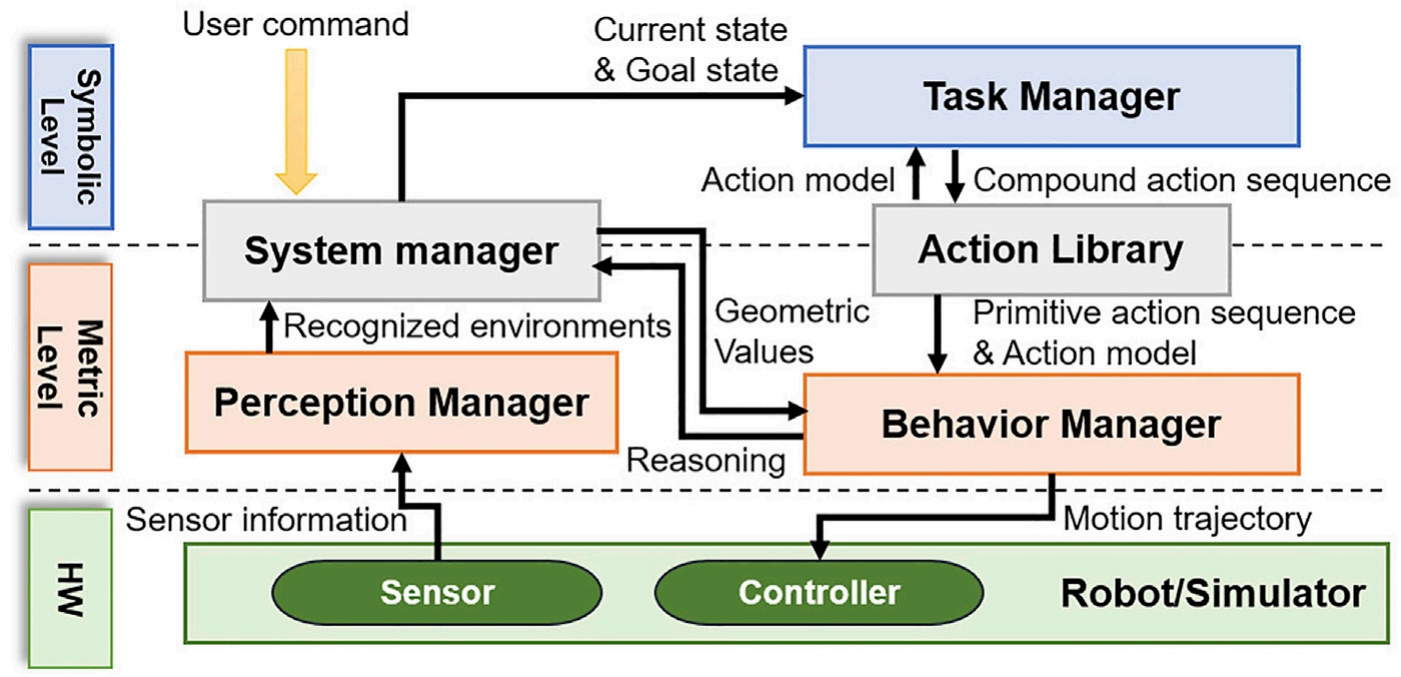
Overview of the proposed task and motion planning system.

In this paper, a CTAMP system is proposed for the service robot to provide various services in object manipulation (such as the handover of objects or pouring drinks). The system we propose consists of five modules: perception manager, system manager, action library, task manager, and behavior manager. Figure 1 shows the system structure. Symbolic-level planning usually involves abstract reasoning, and there is a task manager module for task planning. At the metric-level, perception managers and behavior managers perform geometric calculations. Between the two levels, the system manager and the action library act as interfaces between the other modules. The perception manager recognizes the surrounding environment and objects by using a lidar or a vision sensor attached to the robot. Finally, the perception manager periodically sends all the recognized information to the system manager.

The system manager stores and updates all information received from the perception manager and returns the corresponding geometric value when requesting specific information from other modules. When a command from the user to perform a manipulation task is entered into the system manager, the system manager infers the goal state from the command. Subsequently, the system manager uses the geometric reasoners managed by the behavior manager to infer the current state with the object information recognized by the perception manager. The system manager converts the inferred goal and the current states into the format required by the task manager, and it requests the task planning from the task manager.

The action library is a module that acts as an interface between the task manager and the behavior manager. The action library defines the action model for the manipulation tasks, and it transfers the action model to the task manager to support the task planning. To enable geometric reasoning for an action sequence that is the result of task planning, the action library transfers the action constraints and the action sequence to the behavior manager.

The task manager is a module that calculates the sequence of actions for performing manipulation tasks using a PDDL-based task planner. To plan tasks, the task planner requires input data of the planning algorithm. This includes not only the current state and the goal state, but also action information modeled in the task-planning language. Because the models of actions that a robot can perform are defined in the action library, the task manager extracts the action models from the action library before the task planning. When the task manager receives a request for task planning from the system manager, it plans the task using the state information, which is received from the system manager and the action library model.

The behavior manager manages the algorithms that can perform various geometric calculations so that the actual robot can perform actions. First, geometric algorithms include the motion planners that generate the joint trajectory. The motion planners can verify whether an action is feasible by creating a collision-free joint trajectory. The behavior manager receives the successfully created action sequence from the task manager, and it receives the action model from the action library. For each action of an action sequence, the behavior manager calls the specific motion planner specified in the action model to calculate the joint trajectory, and it transfers the calculated path to the robot controller so that the robot moves according to the trajectory. The geometric algorithms also include geometric reasoners. The behavior manager uses geometric reasoners (such as the condition-checker for grasping) to determine whether the robot has successfully completed the action. Moreover, the behavior manager can help the robot to replan the action because it can determine the success of action by using a geometric reasoner. If the motion trajectory is not created before performing the action, or if the action fails along the trajectory, the behavior manager requests replanning from the system manager. Thus, the system manager updates the current states. Finally, the task manager performs the task planning again.

The above process is repeated until all actions of the action sequence are successfully performed. In addition, when the system manager requests reasoning from the behavior manager to create the predicates of current states before the task planning, the behavior manager infers accessibility of objects, obstacle relocate positions, etc., and returns the results to the system manager.

## 3 Action Library

In the combined CTAMP system proposed in this paper, the action library not only defines the action model to help with the task planning but also acts as an interface between the task planner and the motion planner. To define the action model, the action library classifies actions into compound actions and primitive actions. Moreover, it defines the network structure, where primitive actions are composed of compound actions. In addition, the action library defines which motion planners are needed for each primitive action and what input information is required by the motion planner to generate motion trajectories. We implement a PDDL-based task planner, which requires the action models defined in the planning language as input information.

To model the actions, a difficult problem must be solved: how to express each action specifically. As an example of a manipulation task, assume that there is an obstacle (e.g., juice box) that prevents you from grasping the desired object (e.g., milk box) by hand. If we simply define the action and plan the action sequence, we can reach the goal state by performing the following actions in order.

(1) open_hand (hand)
(2) hold_object (hand, juice)
(3) relocate_object (hand, juice)
(4) release_object (hand, juice)
(5) hold_object (hand, milk)

In the above action sequence, the robot opens its hand, grasps the juice that’s blocking target object, moves it to another place, releases the juice box, and grasps the target milk box. Each action has a clear precondition and a postcondition for causal reasoning. The action of moving the obstacle can be called before the action of grasping the target object. This is because the target object can be blocked by the obstacle, which must be cleared before the action is performed. To perform these actions in practice, it is necessary to prepare a motion planner to create a collision-free path for each action. With additionally defined actions for manipulation tasks, there is a problem of designing additional motion planners.

To solve this problem, we divided the *hold_object* and *relocate_object* actions into the several different actions to express various manipulation tasks with a small variety of actions. We defined an action that can be divided into different actions as a compound action and an action that cannot be further divided as a primitive action. If the above action sequence is expressed in only a few primitive actions, it can be expressed as follows.

(1) open_hand (hand)
(2) move_arm (hand, juice)
(3) close_hand (hand)
(4) move_arm (hand, juice)
(5) open_hand (hand)
(6) move_arm (hand, milk)
(7) close_hand (hand)

The action of holding an object was divided into the action of closing the hands after moving the arm near the object, and the action of moving the obstacle was changed to the action of moving the arm. The action sequence became longer, but the types of actions that form the sequence were reduced from four to three. The second action sequence can be expressed as a combination of several primitive actions, which has the advantage of reducing the number of planners required to verify the action. However, from the perspective of a task planner that performs causal reasoning between actions, the task cannot be planned because the action of moving the arms and closing the hand does not result in the obstacle being removed. To solve this problem, we define the actions in the action library as a network structure of compound actions and primitive actions that constitute a compound action. In addition, the action library describes the motion planner and the action constraints required for each primitive action. Hence, the variables expressed by the symbol for the task planning can be converted to geometric values. The following sections describe how the action model is defined.

### 3.1 Library Definition

To describe the action models in the action library, a script was written using the PDDL style and syntax (Aeronautiques et al., 1998). As a result, the editor is more convenient, and the existing PDDL planners can be easily applied. Definition 3.1 refers to the elements constituting the action library, and Definition 3.2 and Definition 3.3 refer to the elements constituting the compound action and the primitive action, respectively.

Definition 3.1 (Action library). The action library *L* is a tuple <D,T,P,A> where *D* is the set of the robot components, *T* is the set of variable types, *P* is the set of predicates, and *A* is the set of actions.

As an example of *D*, a humanoid service robot’s component set *D* is {*Arm, Gripper, Mobile*}. Hence, we know the robot components needed to perform the actions defined in the action library. Next, *T* is intended for task planning and is used to convert symbolic variables into geometric variables during motion planning. For example, *T* can be expressed as {*Object, Position}*, where *Object* is a variable containing a 3D-shape model, size (height, width, and depth), and class, and *Position* is a simplification of the three-dimensional (x, y, z) coordinates. Moreover, *P* is a set of predicates with Boolean-valued functions, which are set to true or false during the reasoning when the task planner creates a plan. Finally, *A* is a set of actions modeled in the PDDL language, such as *A* = {*a*_1_, … , *a*_*k*_, $a_{1}^{\prime }$ , …, $a_{j}^{\prime }$ }.

Definition 3.2 (Compound action). Each compound action element *a*_*i*_ ∈ *A* is represented by a tuple *<* *param*(*a*_*i*_), *pre*(*a*_*i*_), *eff*(*a*_*i*_), *prim*(*a*_*i*_)*>*, and *param*, *pre*, and *eff* are the same as parameters, precondition, and effect in action definition of PDDL. The *prim* is a low-level primitive action set that composes a *a*_*i*_.

Definition 3.3 (Primitive action). The primitive action *a*′ which is partial plan of compound action is a tuple *<* *param*(*a*′), *pre*(*a*′), *eff*(*a*′), *req*(*a*′)*>* where *req* is a subset of requirements of the action. The *req* is consist of *hardware*_*group* and *planner* sets, *planner* represents the motion planner needed to verify whether *a*′ is feasible, and *hardware*_*group* represents the components of the robot platform included in *D* needed to perform *a*′.

### 3.2 Action Decomposition

**FIGURE 2:**
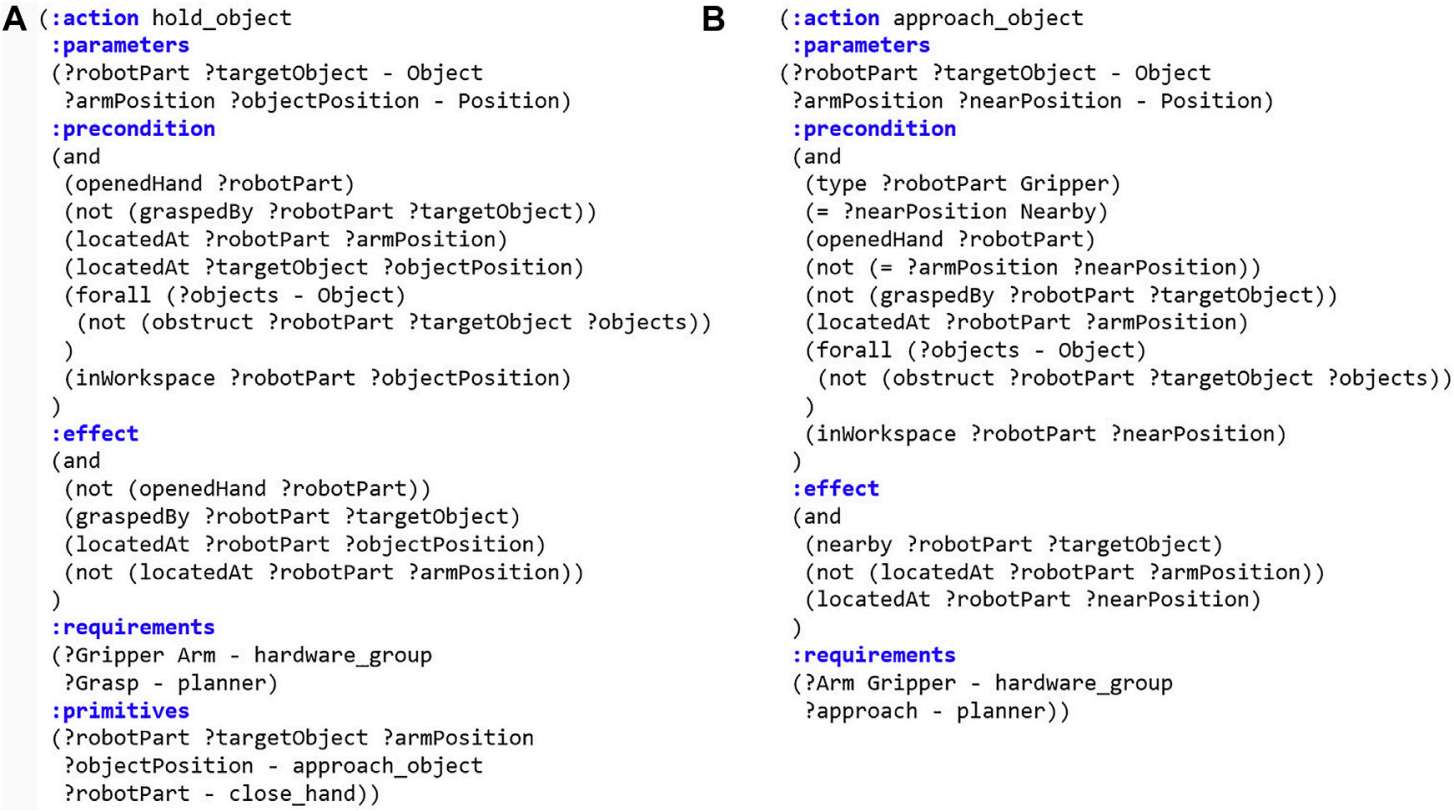
PDDL representation of actions. **(A)** Compound action *hold_object*. **(B)** Primitive action *approach_object*.

Figure 2 shows the two actions described according to the method defined in Section 3.1, where *hold_object* is a compound action and *approach_object* is a primitive action. When the robot plans the task to grasp an object, for example, the action *hold_object* is included in the sequence of actions. Then, *hold_object* is decomposed into *approach_object* and *close_hand*, as specified in (: *primitives*), and is transferred to the motion planner. When the behavior manager plans motions, it should call a primitive action-specific planner described in (: *requirements*) of *planner* from the action model, and it also transfers the input variables to the planner.

**FIGURE 3:**
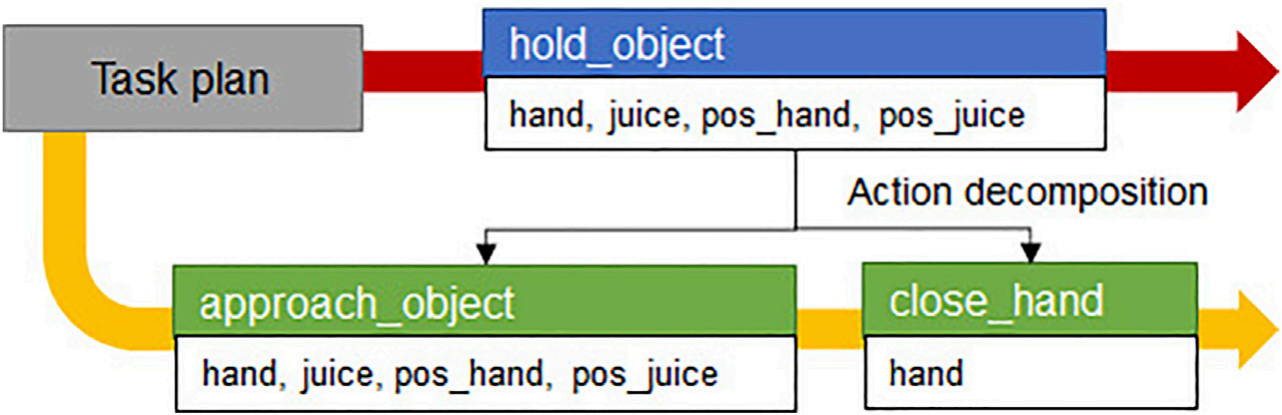
Example of action decomposition. The red arrow is the first planned compound action sequence from task manager and the yellow arrow is the primitive action sequence after the action decomposition.

For a compound action *a*_*i*_, a primitive action set *prim*(*a*_*i*_) = {*P*_1_, … , *P*_*j*_} and *P*_*j*_ = {*p*_1_, … , *p*_*k*_} = $param\left(a_{j}^{\prime }\right)$ where *P*_*j*_ is a parameter set of primitive action $a_{j}^{\prime }$ that must be performed *jth* to perform *a*_*i*_. For example, hold_object(hand, juice, pos_hand, pos_juice) is decomposed into approach_arm(hand, pos_hand, pos_juice) and close_hand(hand) before performing the motion planning as shown in Figure 3.

In this paper, the motion planner specialized in *approach_object* action is defined as an approach-motion planner that creates a joint trajectory to approach and grasp the object. When converting a compound action into primitive actions, the action library should inform the behavior manager which parameters in the compound action correspond to those in primitive actions. In the (: *parameters*) syntax of the *hold_object* action model, the action parameters are the robot component *robotPart*, target object *targetObject*, current position of the robot arm *armPosition*, and the position of object *objectPosition*. The robot component *robotPart*, target object *targetObject*, robot arm initial position *armPosition*, and target object’s nearby position *nearPosition* are parameters of the *approach_arm* action.

## 4 Task Manager

**FIGURE 4:**
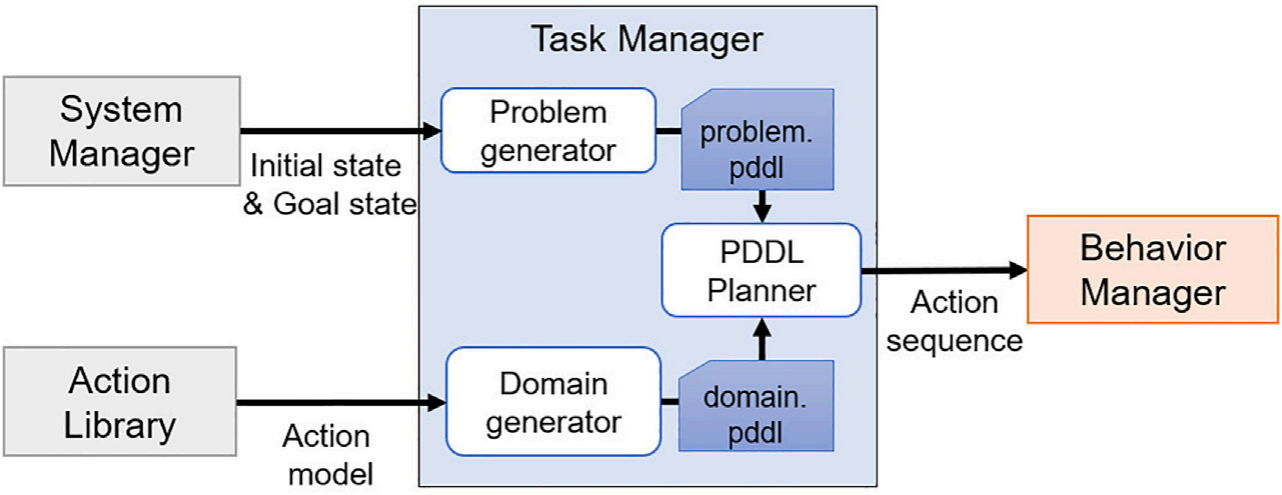
Diagram of the task manager.

The task manager is a module to automatically plan tasks, which was implemented as shown in Figure 4. The task manager plans tasks using PDDL, and the PDDL task planner needs two script files for planning: Domain. pddl and Problem. pddl. The problem generator generates problem. pddl by receiving the current state and the goal state from the system manager, and the domain generator generates domain. pddl by receiving the action model from the action library. When task planning is successfully executed using two script files, an action sequence is obtained as a result, and it is transferred to the behavior manager.

### 4.1 Problem Generator

The problem generator receives current and goal states from the system manager and all objects recognized by the perception manager. Then, received information is converted into the PDDL syntax and stored in problem. pddl.

### 4.2 Domain Generator

The domain. pddl file contains the actions that the robot can perform, predicates, and object information. These contents are already defined in the action library, and the set of actions that can be performed is *A*, the predicates are *P*, and the object information is *T*. The domain generator reads only what is needed for the task planning from the action library and saves it to the script files, excluding *prim* and *req* of *A*. In addition, the domain generator only brings the actions from the action library that can be performed by comparing the components of the robot with the *hardware*_*group* defined in *A*. By doing this, the actions that cannot be performed on the current robot platform are excluded from *A* to prevent including them in an action sequence.

When script files are generated by the problem generator and the domain generator, the task planning is performed using the PDDL planner. The task planning uses a classic fast-forward algorithm (Helmert, 2006) that uses the state search method to obtain an action sequence.

Algorithm 1 Motion generator algorithm.

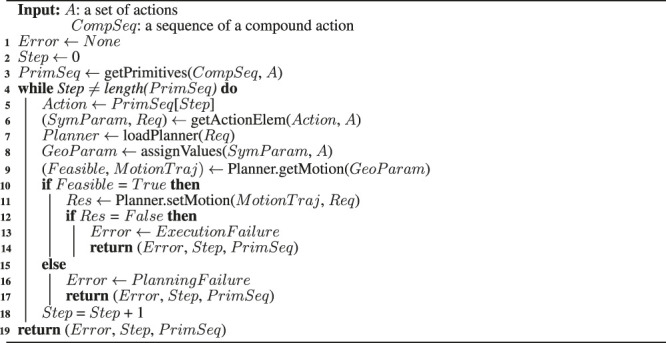

## 5 Behavior Manager

**FIGURE 5:**
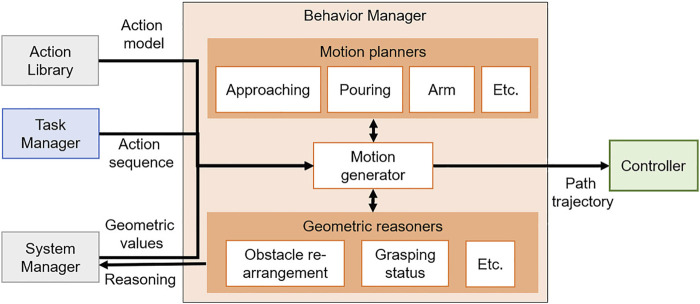
Diagram of the Behavior manager.

To plan motions for primitive actions, the behavior manager manages modularized motion planners and reasoners and acts as an interface between the modules. The behavior manager is implemented as in Figure 5. Hence, the actual robot can perform the actions obtained as a result of the task plan in order. When a compound action sequence comes from the task manager, the motion generator converts the compound action sequence into a primitive action sequence by using the relationship between actions in the action library. For each action in the converted primitive action sequence, the motion generator verifies whether it is possible to create a collision-free path capable of performing the primitive action using the motion planner and the reasoners managed by the behavior manager. The specific method is described in Section 5.1. When the collision-free path is generated successfully, the motion generator transfers the path to the robot controller so that the robot moves along the path. If the motion planner fails to generate a path or the robot fails to perform a primitive action, the motion generator requests replanning from the system manager to ensure that all actions are performed successfully.

In the following sections, we explain how the behavior manager creates motion for an action and enables replanning in the CTAMP system.

### 5.1 Motion Generation

The motion generator has the role of calculating a joint trajectory of the action using the motion planner modules. Moreover, it converts the compound action sequence received from the task manager to the primitive action sequence before the motion planning. The motion planner modules managed by the behavior manager return the calculated motion using each algorithm when input values for motion planning are given. To create a joint trajectory, the motion generator automatically calls a motion planner that is specific to the current primitive action among motion planners. At this time, the motion generator must know what motion planner should be called and what inputs are required by it. Moreover, it assigns geometric values because the actions of the transformed action sequence are expressed only by abstract symbols.

Because motion planners and inputs that are necessary for the motion planning of primitive actions are already defined in the action library, and all geometric values obtained from the perception module are stored in the system manager, the motion generator takes the necessary information from them. When inputs are given to the called motion planner, the joint trajectory of the primitive action is generated and transferred to the controller, and the motion generator repeats all actions within the action sequence, allowing the robot to perform the action. Algorithm 1 shows the process of automatically generating the joint trajectories for all actions and transferring it to the controller using the compound action sequence and the action model by the motion generator.

Before the motion planning, the motion generator receives the compound action sequence *CompSeq* provided by task planning and the action set *A* modeled in the PDDL language from the task manager and the action library, and it uses the getPrimitives() function to convert *CompSeq* into primitive action sequence *PrimSeq* [line 3]. Through the *while* loop, for each primitive action in *PrimSeq*, the motion generator checks the current primitive action to be performed and receives the information related to it from the action set *A* [lines 5-6]. In *A*, necessary conditions of actions for task and motion planning are defined, and getActionElem() function brings only the action parameters *SymParam* and requirements *Req* of the action required for motion planning. The necessary planner for the motion planning of the action and the robot component performing the action are defined in *Req*, and the loadPlanner() function takes it and calls the specific planning module *Planner* among the modules managed by the behavior manager. The assignValues() function is a function that receives geometric values that correspond to the action parameter *SymParam* expressed only by symbols required by the motion planner module. Then, the geometric values stored in the system manager are returned and stored to *GeoParam*. Suppose that the current *Action* is *approach_object (left_hand, obj_juice, pos_left_hand, pos_juice)*, and motion planning should be performed on this. The *approach_object* action is to take an approach to an object before grasping it, and the specific motion planner of this action is defined as the approaching motion planner in *A*. The approach-motion planner in the behavior manager we have implemented needs the Unified Robot Description Format (URDF) file containing the geometry information of the robot, the position and pose of the object, and the 3D mesh file for planning. Because these geometric values are managed and stored in real-time by the system manager, the motion generator can receive values corresponding to the motion planner’s input from the system manager. An action parameter *obj*_*juice* is an instantiated symbol of *Object* in *SymParam*, and when a value of *obj*_*juice* is requested from the system manager, 3D shape information (including the mesh file and the size of the target object) is returned. For the *left*_*hand*, in the same way, the motion generator will receive URDF for the robot.

For *pos*_*juice* and *pos*_*left*_*hand*, the motion generator will receive geometric values of the three-dimensional position (x, y, z) and orientation (x, y, z, w) of the juice box and the left hand’s end-effector, respectively. When *GeoParam* is transferred to getMotion() function of planner module, a collision-free path is planned. If the path is created successfully, *MotionTraj* and *Feasible* gets *True* value [lines 9–10]. When motion planning is finished, *MotionTraj* is transferred to the controller via the setMotion() function to allow the robot to move along its path and repeat the above steps for the following actions [line 11]. This time, *Req* is also used as a variable for the setMotion() function, and because the *hardware*_*group* is defined in *Req*, the motion generator can transfer the joint trajectory to a specific controller. The motion generator can automatically call the motion planning module corresponding to the primitive action and transfer the necessary information for the motion planning, as shown in Algorithm 1. Therefore, by adding the modularized state-of-the-art motion planner into the behavior manager and defining the action model in the action library for a specific action, the robot can perform a manipulation task using various actions. Moreover, because the geometric information of the current robot is obtained from the URDF file, the motion generator algorithm can be applied independently of the hardware if the robot platform is defined in URDF.

We have implemented the motion planner modules to support several motions that can be replaced with state-of-the-art algorithms in the behavior manager: arm motion, approaching motion, gripper motion, pouring motion, and handover motion. The motion planning algorithms for specific actions are independent research subjects. In this study, we do not focus on the implementation of the optimized or efficient motion generation algorithms. Instead, we simply implement a motion planner that returns a motion when input is given.

#### 5.1.1 Arm Motion Planner

The arm motion planner module calculates the motion trajectory for moving the robot arm to the target pose from a current pose. The planner obtains kinematic information from the robot URDF, and creates a collision-free path with the 3D pose of the robot hand’s end-effector and recognized objects.

#### 5.1.2 Approaching Motion Planner

The approach-motion is a motion that creates a path so that the robot moves its arm in a position before it grasps an object. The approach-motion planner that we have implemented receives the following inputs: robot’s URDF and 3D mesh file such as the STL format of the target object, 3D positions, and poses of the objects. Using the target object shape, the approach-motion planner generates several pose candidates that become force-closure when the robot gripper is closed and grasps the target object and returns a successfully generated path by calculating inverse kinematics (IK) whether there is a collision-free path from the current position of the robot’s arm to each candidate position.

#### 5.1.3 Gripper Motion Planner

The gripper motion planner creates a gripper’s joint trajectory to grasp or put an object. The *close_hand* action can grasp the object by simply closing the gripper joints because the *approach_object* action to take the pose before grasping the object is performed first by the task planning, and force-closure is calculated at this time. Therefore, we used only two predefined joint angles to open and close the gripper.

#### 5.1.4 Pouring Motion Planner

**FIGURE 6:**
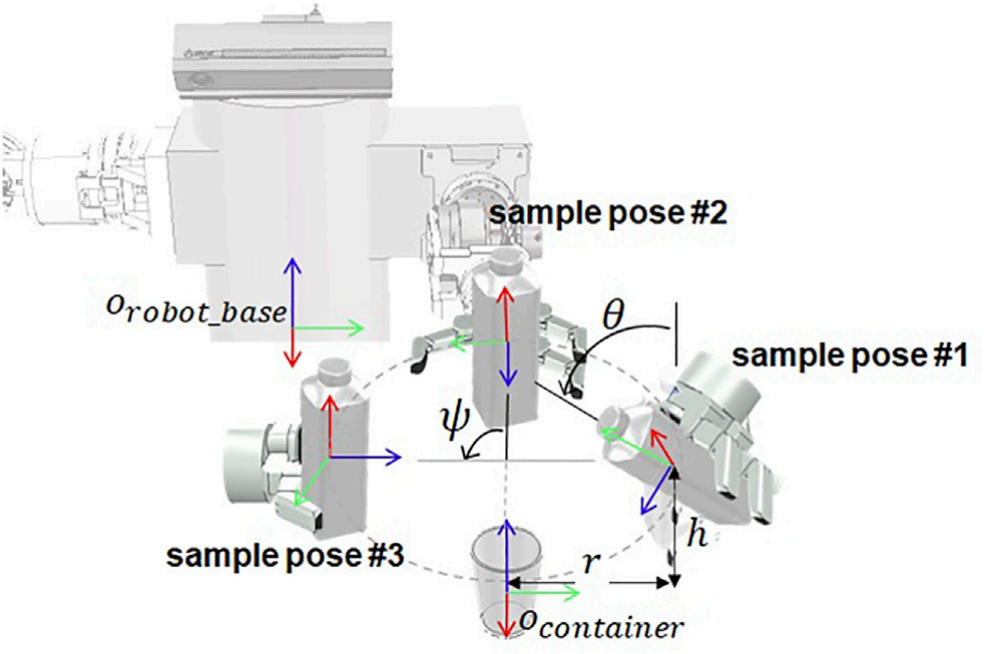
Sample poses of the robot end-effector based on the robot base coordinate system, which are used to calculate the pouring motion.

The pouring motion is a motion that pours a container containing a beverage into another empty container, which is necessary to perform a manipulation task, such as providing a drink. Pouring motion planning has many considerations such as fluid flow for the stable pouring, and there are studies using force sensors or algorithms that recognize the affordance of an object (Pan et al., 2016; Tsuchiya et al., 2019). We simplify the pouring action so that the beverage is not considered. We define in advance several end-effector’s sample pose *P*_*sample*_ to pour the beverage, as shown in Figure 6, which are located by distance *r* in the horizontal *x*-*y* direction and by *h* in the vertical *z*-direction from the central coordinates of the empty container and are rotated to *z*-axis. The pouring action is considered successful if it is possible to take an inclined posture by angle *θ* from the sample position. As a result, a pouring motion trajectory *ζ*_*pour*_ from the current end-effector’s pose is

Ppre=Rz,ψtcb01⋅I3r0h01⋅Teb (1)

$$P_{sample}=P_{pre}\cdot \left[\begin{array}{cc} R_{y,\theta } & 0\\ 0 & 1 \end{array}\right]$$ (2)

$$\zeta_{pour}=\zeta_{pre}+\zeta_{tilt}$$ (3)

where *P*_*pre*_ is the pose of end-effector before tilting an object in the sample position, tcb is 3 × 1 translation matrix from robot base to container, *R*_*z*,*ψ*_ is 3 × 3 rotation matrix around the *z*-axis by *ψ* degrees, Teb is 4 × 4 transformation matrix from robot base to end-effector of the gripper and *ζ*_*pre*_ is joint trajectory for approaching motion from *P*_*eef*_ to *P*_*pre*_, *ζ*_*tilt*_ is joint trajectory for tilting motion from *P*_*pre*_ to *P*_*sample*_ and calculated with arm motion planner. In this paper, we defined three sample poses to pour the beverage with *ψ* = [ − 90°, 0°, 90°]. For this purpose, the pouring motion planner receives the following inputs: the pose of the target container and the robot end-effector.

#### 5.1.5 Handover Motion Planner

**FIGURE 7:**
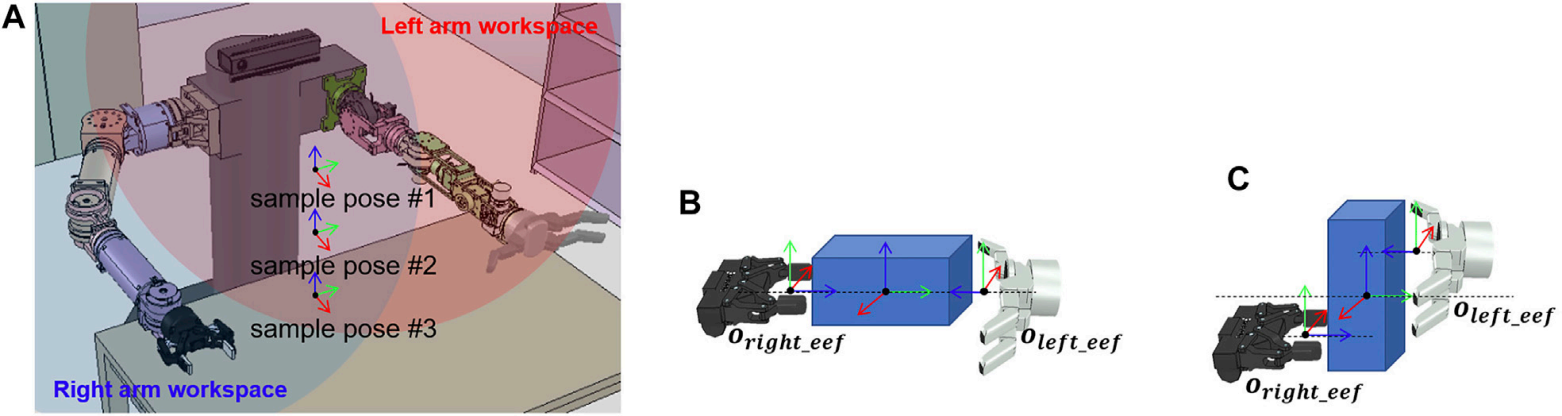
Sample poses of the object and the robot end-effector, which are used to calculate the handover motion. **(A)** Sample poses of the object located in the dual-arm workspace. **(B)** Sample pose of the left and right end-effectors when the object is lying down. **(C)** Sample pose of the left and right end-effectors when the object is standing.

Handover motion refers to a motion in which the robot moves an object into the workspace of both robot arms to pass the object held by one hand to the other hand. To pass the object, we calculate the end-effector position to grasp the object with the rest of the robot’s hands after moving the grasped object to the position within the workspace of both arms. We simplify the calculation to find the position for passing the object. As shown in Figure 7A, the sample poses of the object within the workspace of both arms are previously defined. The poses of the end-effector for grasping the object with both hands is fixed in that sample positions depend on the shape of the object and the hand that transfer the object as shown in Figures 7B, C. When an object is placed in a sample position, and there is an IK solution that satisfies the pose of both hands, the planner returns a motion trajectory to move the robot hand, grasping the object to the sample position as a result of planning.

### 5.2 Replanning

If the robot successfully performs all actions in the primitive action sequence, the task is finished. However, if the path is not created before the action is performed, or the action is not successfully performed after the path is created, we update the current state to reach the goal state and encourage other actions to be performed through the replanning. If the path is not created, it occurs because the task planner cannot make geometric inferences.

**FIGURE 8:**
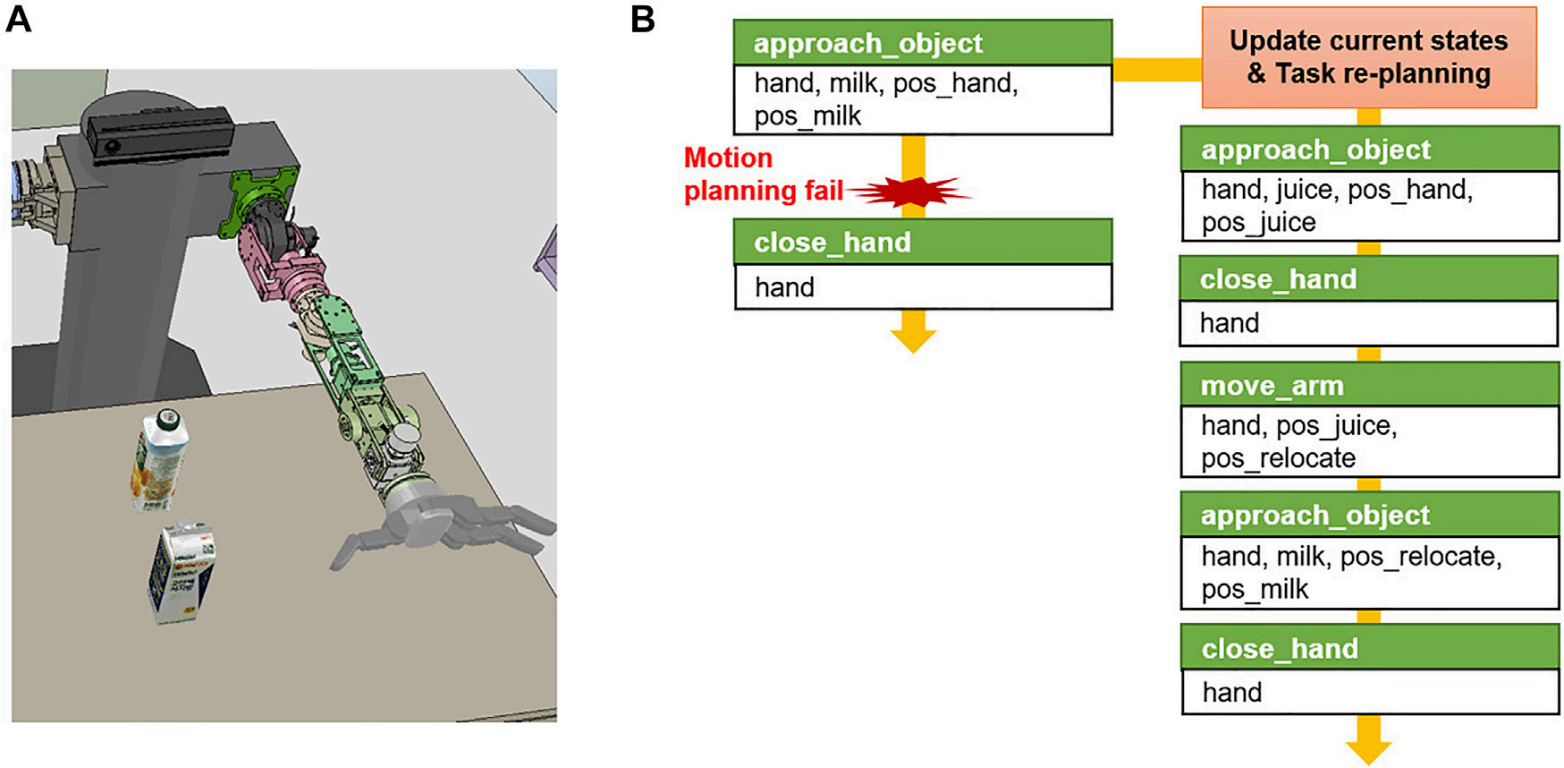
Example of task re-planning in the pick and place task domain. **(A)** Initial simulation environment scene. **(B)** When the robot failed to motion planning to execute *approach*_*object* action, the task manager re-plans primitive action sequence.

For example, in the case of picking a task in which a target object *milk* is obstructed by the obstacle object *juice* as shown in Figure 8A, the action of removing the obstacle is included in the action sequence only when the state that where target object is being blocked is known through the geometric reasoning before the task planning. Otherwise, the task planner is not aware of the current state where the obstacle exists, and it performs the initial task plan as shown in Figure 8B. Hence, only the action of grasping the target object is included in the action sequence, but the motion planner does not generate a path for the robot arm to reach the target object because of the obstacles. In this case, the motion generator returns Error that the motion planning has failed, as shown in line 16 of Algorithm 1. When the system manager receives an error regarding a failure, it updates the current states with the reasoners in the behavior manager and requests to the task manager for acquiring the re-planned action sequence as shown in Figure 8B.

In this study, we have implemented the reasoner for obstacle rearrangement (Lee et al., 2019) for this purpose. Obstacle rearrangement reasoner is an algorithm that uses the vector field histogram+ (VFH+) to verify the accessibility of the target object. If the object is not accessible, this reasoner calculates the order and position of relocating the obstacles. The system manager converts the reasoning results into a PDDL predicate format, adds it to the current state, and requests the task manager to replan. In the above example of object picking, the reasoner infers that the target object is blocked by the obstacles, and the system manager adds the predicate *obstruct (gripper, targetObject, obstacle)* to the current state to replan the task manager from the updated current state. Therefore, the robot can respond to changes in the environment by adding the predicates to the current state and the actions to remove the obstacles to the action sequence.

The failure of performing the action occurs when the position of the recognized object is different from the actual position or if the robot cannot follow the motion trajectory due to the uncertainty of the recognition algorithm or the robot control algorithm. For example, when performing a manipulation task to grasp an object, a path is generated by motion planning with the recognized object position. However, suppose that the object is not grasped by the robot hand at that position due to an uncertainty error. The motion generator determines the success of the action using the reasoners whenever it performs an action using the setMotion() function, such as line 11 of Algorithm 1, and returns the result as *True* or *False* in *Res*.

For this purpose, we have implemented a reasoning module to determine the grasping status. The grasping status reasoner can infer the open/close state of the gripper because it receives information on the joint angles of the robot gripper and the position of the objects, and the criteria for determination of gripper’s open/close conditions is defined. In addition, the grasping status reasoner can be used to infer whether an object is grasped using the distance between the objects and the gripper end-effector and the status of the gripper. If the target object is not grasped and the action fails, the motion generator uses a grasping status reasoner and transfers Error to the system manager. The motion generator returns the cause of failure *Error*, *PrimSeq*, and the last step of action *Step*, as shown in line 19 of Algorithm 1, to remind the system manager which step requires replanning.

The system manager uses the reasoners to update the current states and requests the task planner to resume the task from the step in which the action was successfully performed. The replanning is repeated until the robot performs all the primitive actions. To prevent infinite replanning, we limit the number of attempts.

## 6 System Evaluation

### 6.1 Implementation

The CTAMP system proposed in this study is tested in simulations on Intel G4560 with 16 GB RAM. The simulator is V-REP, and the physical engine is Vortex. We implemented the task planner module using the pddl4j open library (Pellier and Fiorino, 2018) for PDDL-based task planning in the task manager, and we calculated grasping force-closure and IK using moveit (Chitta et al., 2012) and graspit (Miller and Allen, 2004) for the implementation of motion plan modules in the behavior manager. All the modules of the proposed system are implemented in the Robot Operating System (Quigley et al., 2009).

For the experiment, three table-top tasks (object handover, beverage pouring, and obstructed object) are defined, and a virtual environment is implemented. The robot is self-made, having 6-dof manipulator (Choi et al., 2016) with Robotiq 2F-85 and 7-dof manipulator with Barrett hand BH8-282, and the robot base is fixed. Each robot manipulator and gripper is controlled by the controller of the V-REP simulator. The manipulators move through the position control when the joint trajectories are obtained from the motion planner, and the grippers move the joints through the velocity control according to the open/close commands. The 3D shape information of the objects used in each experimental environment is stored in the system manager, and the position and pose of the objects and the robot are obtained from the simulator in real-time.

### 6.2 Object Handover

**FIGURE 9:**
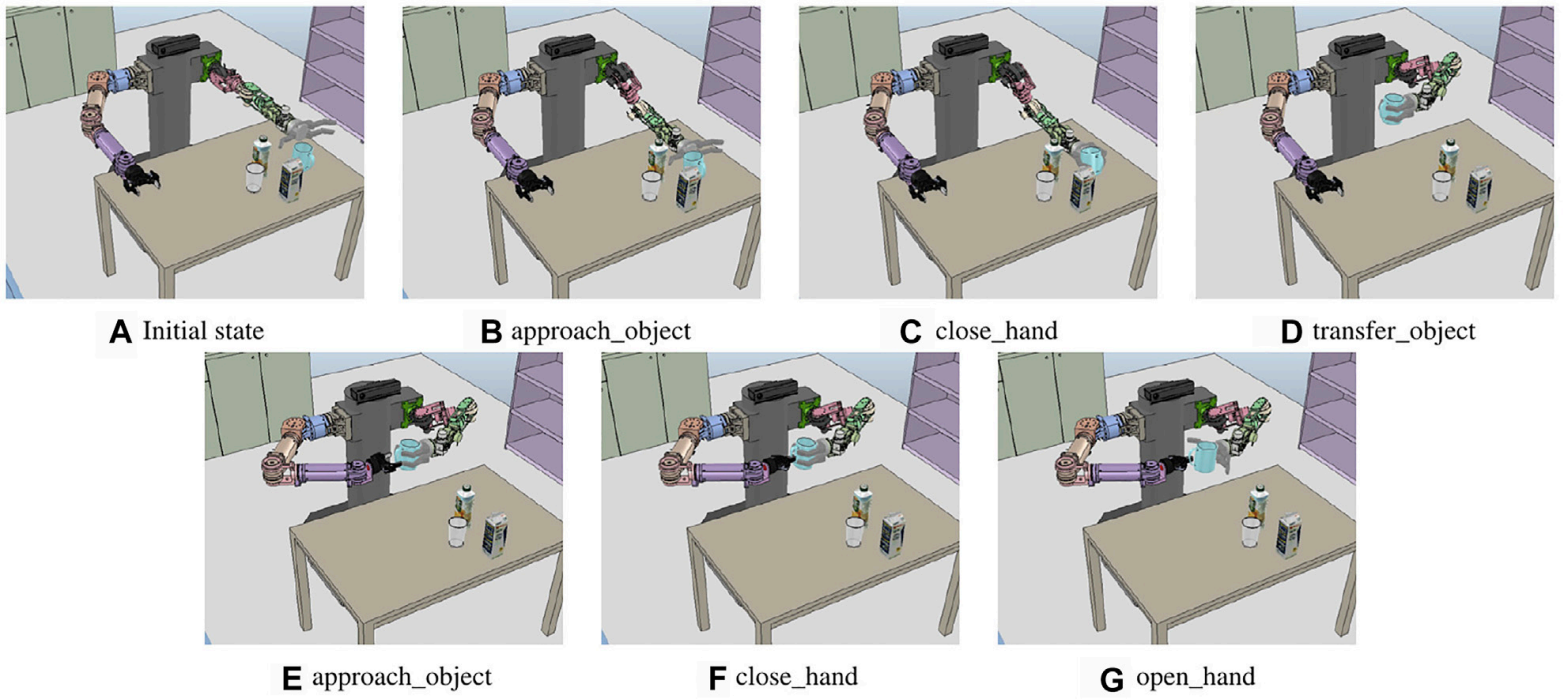
Sequence of the execution snapshot for the object handover problem. **(A)** Initial state of the handover problem. *left*_*hand*, *right*_*hand*, *obj*_*mug*, *obj*_*milk*, *obj*_*juice*, and *obj*_*cup* are recognized and transferred to the system manager. **(B–G)** show the results of performing a primitive action from **(A)**.

The first experiment is shown in Figure 9A. The task is to hand over objects in the environment where a mug, glass cup, juice box, and milk box are placed on the table. All objects are in the workspace of the left hand, and the objects must move into the workspace of the right hand to grasp an object with the right hand. We defined the goal state using predicates *graspedBy(right_hand, obj_mug)* and *¬* *graspedBy(left_hand, obj_mug)* to hold the mug with the right hand in this task.

The initial state is automatically generated using the predicates by the system manager, and the system manager first generates a predicate for a recognized object position. The 3D coordinates where all recognized objects are located are represented by the *locatedAt* predicate. Next, the system manager generates additional predicates using the reasoner. The system manager first calls the obstacle rearrangement reasoner, transferring the position of the robot and the object from the behavior manager, and it starts to infer whether the object is accessible. All the objects accessible by the left and right hand are represented by the *inWorkspace* predicate. The *openedHand* predicates are generated from the results of the grasping status reasoner. Below is a list of the initial and the goal state predicates generated by the system manager prior to planning the task for the handover problem.

Initial state:
*openedHand*(*left*_*hand*), …
*locatedAt*(*obj*_*mug*, *pos*_*mug*), …
*locatedAt*(*left*_*hand*, *pos*_*left*_*hand*), …
*inWorkspace*(*left*_*hand*, *pos*_*mug*), …
Goal state:
*graspedBy*(*right*_*hand*, *obj*_*mug*),
*¬graspedBy*(*left*_*hand*, *obj*_*mug*)

**FIGURE 10:**
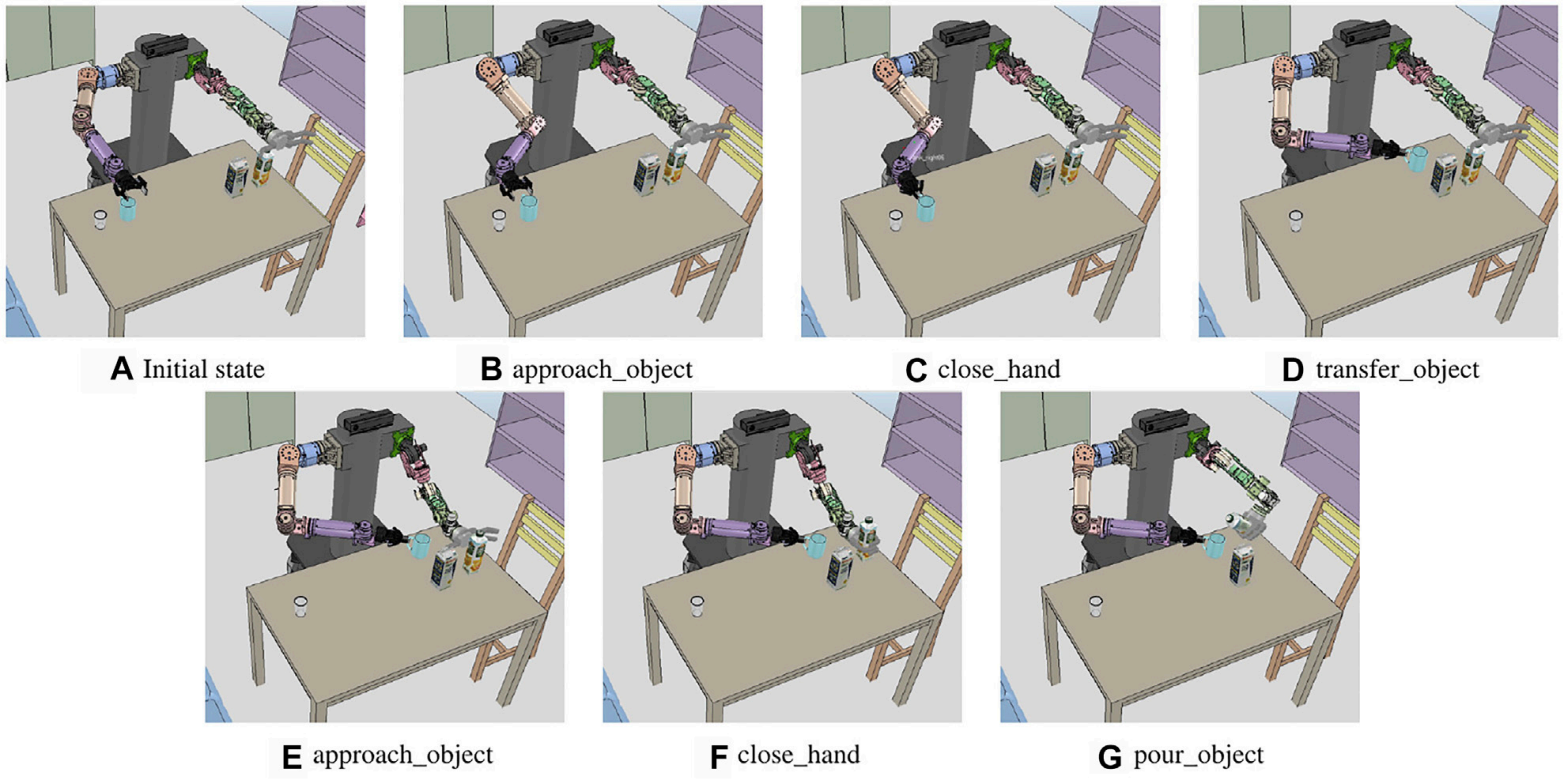
Sequence of the execution snapshot for the juice pouring problem. **(A)** Initial state of the pouring problem. *left*_*hand*, *right*_*hand*, *obj*_*juice*, *obj*_*milk*, *obj*_*cup*, and *obj*_*mug* are recognized. **(B–G)** show the results of performing each primitive action.

Figure 10A shows the sequence of compound actions obtained from the task planning and the converted sequence of primitive actions before the motion planning with the above predicates. Because the robot base cannot move, the compound action sequence, which is the result of the task planning, includes grasping the cup with the right hand after moving it within the workspace of the right hand using the left hand. The *hold*_*object* is divided into *approach*_*object* (approaching the arm to the object) and *close*_*hand* (grasping the cup by closing the robotic hand). In the case of *approach*_*object* action, the motion generator receives the corresponding geometric values from the system manager, and *left*_*hand* is assigned from the robot URDF, *obj*_*mug* is assigned from the mesh file of the mug, *pos*_*left*_*hand* and *pos*_*mug* are the position and pose of the current left end-effector and the mug.

The motion generator calls the approach-motion planner and inputs the assigned values to create a path for the posture before grasping the mug, as in Figure 9B. The *handover*_*object* action is divided into *transfer*_*object* (move the cup with the left hand) and *hold*_*object* (hold the object with the right hand). The handover motion planner is called to perform *transfer*_*object*, and it calculates the path to move the arm by selecting the candidate position *pos*_*handover* to hold the mug with both hands, as in Figure 9. The calculated joint trajectory is transferred to the controller. Hence, the robot moves the cup by moving the left arm, as shown in Figure 9D, and performs *approach*_*object* action with the right hand, as in Figure 9E. Figures 9B–G show the result of each action of the primitive action sequence. As a result, the mug is grasped by the right hand of the robot.

### 6.3 Beverage Pouring

**FIGURE 11:**
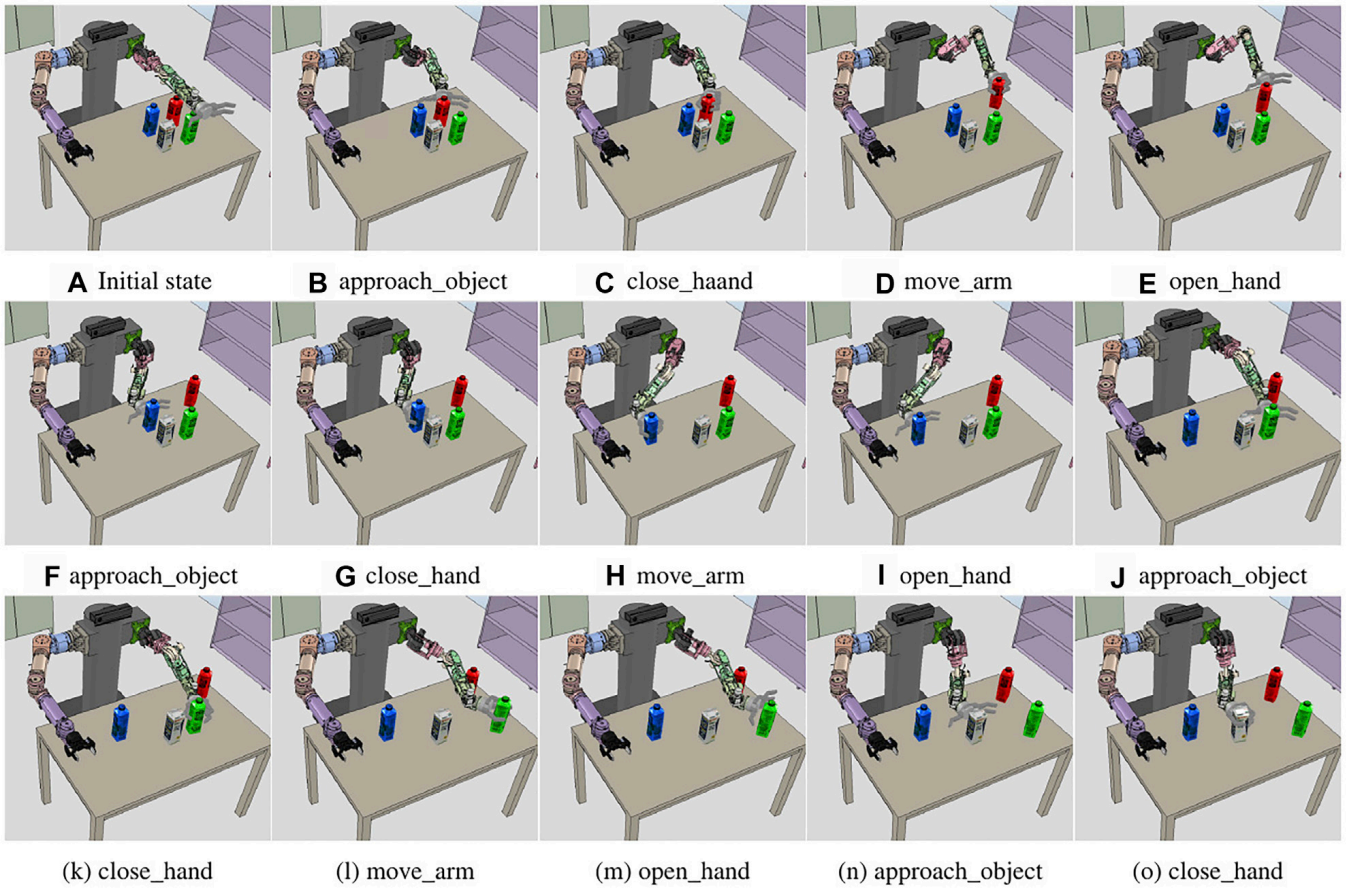
Sequence of the execution snapshot for the obstructed milk box picking problem. **(A)** Initial state of the experiment. *left*_*hand*, *right*_*hand*, *obj*_*milk*, *obj*_*redjuice*, *obj*_*bluejuice*, and *obj*_*greenjuice* are recognized. **(B–O)** show the results of performing each primitive action.

The second experiment is dedicated to the problem of pouring a drink into an empty container. The experimental environment includes cups in the workspace of the right hand and drinks in the workspace of the left hand, as shown in Figure 11A. The goal state predicate is defined as *inContGeneric*(*obj*_*mugobj*_*juice*), indicating that the drink is in the cup. As in the handover problem, the system manager creates predicates for the recognized object position, gripper status, and accessibility between the objects. It defines the states as follows.

Initial state:
*openedHand*(*left*_*hand*), …
*locatedAt*(*obj*_*mug*, *pos*_*mug*), …
*locatedAt*(*left*_*hand*, *pos*_*left*_*hand*), …
*inWorkspace*(*left*_*hand*, *pos*_*juice*), …
*inWorkspace*(*right*_*hand*, *pos*_*mug*), …
Goal state:
*inContGeneric*(*obj*_*mug*, *obj*_*juice*)

Figure 10B shows that the result of the task planning is to move the position of the cup with the right hand and move the juice box with the left hand to the position for pouring it to the cup. To perform the *transfer*_*object* action, the motion generator used the handover motion planner to transfer the motions to the controller: to move the right hand holding the mug to an accessible position and to move the mug into the workspace of both arms, as shown in Figure 11D. Finally, for the *pour*_*object* action, the motion generator obtains the three-dimensional coordinates corresponding to *pos*_*juice* and *pos*_*t*_ *ransfer* from the system manager and obtains the motion trajectories. Hence, the robot moves the right hand from the current position to the cup and tilts the juice box from the pouring motion planner, as described in 5.1. The motion trajectory is transferred to the controller, and the result is illustrated in Figure 11G.

### 6.4 Obstructed Object

**FIGURE 12:**
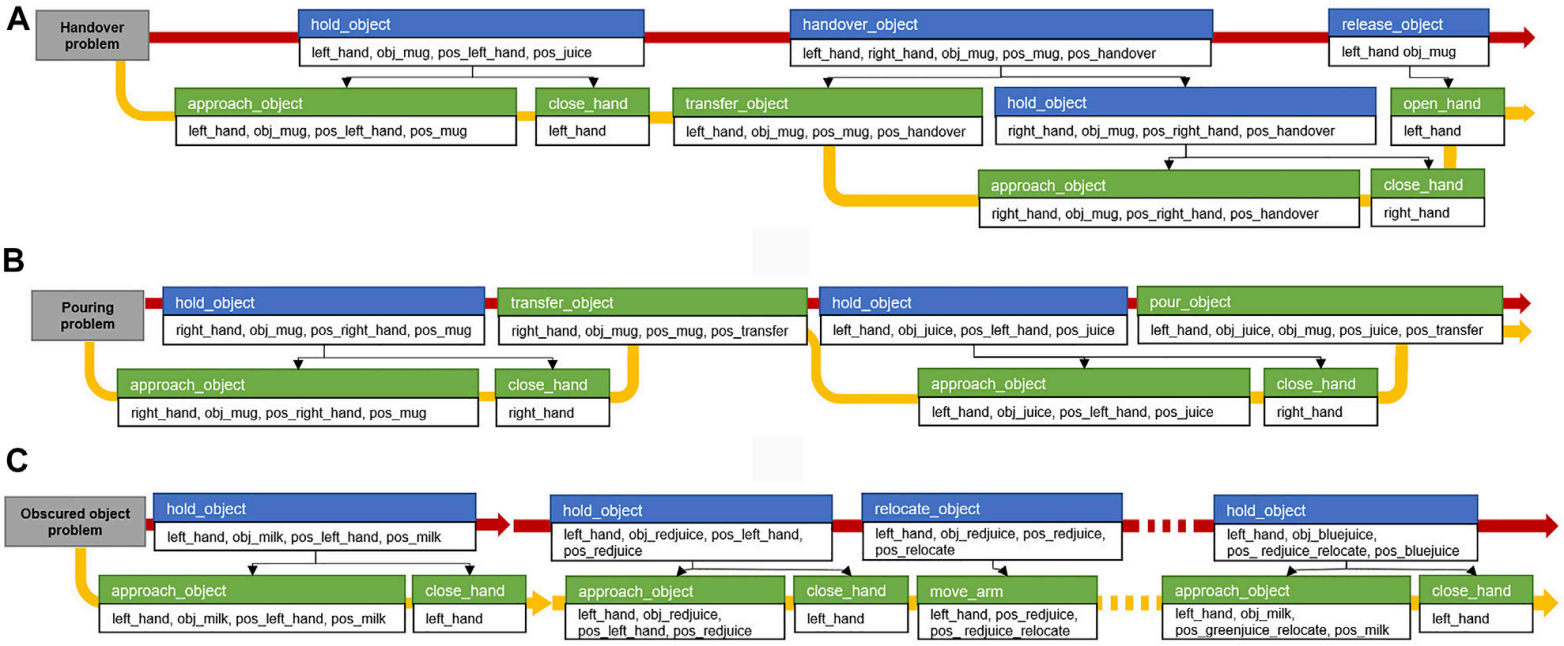
Compound and primitive action sequence from the results of the task planning performed in the three task domains. The blue box is the compound action. The green box is the primitive action. Below are the action parameters. The black arrow indicates the relationship between the compound and primitive actions. The red arrow is the compound action sequence obtained by the task planning, and the yellow arrow is the converted primitive action sequence. **(A)** Action sequence of the object handover problem. **(B)** Beverage pouring problem. **(C)** Obstructed object problem.

The third problem is a situation when the task is interrupted because other objects are placed in the grasping path of the target object. Figure 12A shows the environment corresponding to the initial state, with milk boxes and three colored juice boxes placed around it, preventing the robot from picking up the milk box. To define grasping the milk as the goal state, we used the *graspedBy*(*left*_*handmilk*) predicate. As in the previous experiments, the reasoners are called by the system manager, creating a list of predicates for the initial and goal states, as shown below.

Initial state:
*openedHand*(*left*_*hand*), …
*locatedAt*(*left*_*hand*, *pos*_*left*_*hand*), …
*locatedAt*(*obj*_*milk*, *pos*_*milk*),
*inWorkspace*(*left*_*hand*, *pos*_*milk*)
Goal state:
*graspedBy*(*left*_*hand*, *milk*)

In general, if the system manager calls the obstacle rearrangement reasoner, the predicates are added when geometric reasoners are called before the task planning. As a result of this inference, juice boxes are obstacles for grasping the milk box. However, in this experiment, the task planning is performed except for *obstruct* predicates in the generated problem. pddl script file, ignoring intentionally colored juice information to confirm the replanning. In Figure 10C, the first *hold*_*object* action is the result of the initial work plan, and it shows that the robot is trying to grasp the milk box directly because there is no prior information that juice boxes are obstacles. However, in the process of the motion generator planning, the arm motion planner fails to create the path of the *approach*_*object* action, which is the first action in the primitive action sequence. This is because the juice boxes are placed on the path. Nevertheless, the arm motion planner transfers the failed result to the motion generator. When the action execution fails, and the motion generator transfers an error to the system manager, the system manager calls geometric reasoners from the behavior manager to update the current state. The obstacle rearrangement reasoner receives the 2D space coordinates and size of the objects as input for the table plane where the objects are placed. As a result, it calculates the order and position in which the obstacles are removed. In the inference process, the size of the gripper is also reflected in the size of the object. In Figure 12I, the green juice box is an obstacle because it is separated from the milk box; however, there is a small interval for grasping it using the gripper. The calculated rearrangement position is stored in the system manager as *pos*_*redjuice*_*relocate*, *pos*_*bluejuice*_*relocate*, and *pos*_*greenjuice*_*relocate* variable with three-dimensional coordinate values for each juice box. The following predicates are updated to the current state.

*locatedAt*(*obj*_*milk*, *pos*_*redjuice*), …
*obstruct*(*left*_*hand*, *obj*_*milk*, *obj*_*redjuice*), …
*inWorkspace*(*left*_*hand*, *pos*_*redjuice*), …

After the second *hold*_*object* action in Figure 10C, the result of the replanned task plan is updated, and the *relocate*_*object* action is added. The *relocate*_*object* action is converted to *move*_*arm* action, and the motion generator transfers the relocate coordinate corresponding to *pos*_*redjuice*_*relocate* obtained from the system manager, and it transfers it with the current position of the left arm end-effector to the arm motion planner to obtain the joint trajectory. Figure 12D shows the result of transferring the path to the controller and moving the arm holding the red juice to the relocated position. The same actions are repeated for the remaining juice boxes to perform the obstacle relocating action. As a result, the target milk box is grasped, as shown in Figure 12D.

The system performance for 50 repetitions in the V-REP simulator for the three experiments is summarized in Table 1 with the average success rate, task planning, motion planning, geometric reasoning, and total operation time. The time of motion planning is the sum of the motion planning times of all primitive actions. The time of geometric reasoning is the sum of the state reasoning times before the task planning and after performing the action. Compared with the time spent in motion planning of primitive actions in the handover experiment and the pouring experiment, the obstructed object experiment required less time for motion planning, although the number of performed primitive actions was greater. This is because a position calculation to move the target object to the workspace of a different arm is done during the motion planning phase in two other experiments, while the calculations of relocated positions are done in advance before the task planning in the obstructed object experiment. However, because the obstructed object experiment performed three actions of relocating obstacles, the time spent on planning and reasoning is shorter, but the time spent on the total operation is longer than in other experiments. For the three experiments, task failure is the case of exceeding the number of replanning times. This occurred when the robot collided with the objects, causing the object to fall down or fall under the table, thus, leaving the workspace of the robot.

**TABLE 1:** Results of each experiment in the V-REP simulation performed 50 times.

Measure	Object handover	Beverage pouring	Obstructed object
Success rate	96%	90%	84%
Task planning	0.43s	0.88s	1.08s
Motion planning	8.74s	9.06s	5.54s
Geometric reasoning	0.76s	0.54s	0.94s
Total operation	29.63s	48.84s	81.01s

## 7 Conclusion

In this paper, we proposed a system using the action library, task manager, and behavior manager for CTAMP. In the action library, the actions that the robot can perform are modeled in a PDDL-based language, and the relationship network between the actions and conditions for motion planning are also defined. Using the action library, the task manager decides the order of the actions by the PDDL-based task planning. The behavior manager shows that the motion planner, reasoner, and necessary conditions to perform each primitive action can be received automatically from the action library to plan the motions. Moreover, the behavior manager calls the modularized motion planners and the reasoners. According to our results, state-of-the-art algorithms can be linked to enable efficient planning and facilitate additional applications with various manipulation tasks. In addition, unlike in previous studies, the robot does not move after all the actions are verified, but it performs each action at the moment it is verified. Whenever an action is performed, the result of the action is inferred, and the states are updated to respond to the changes in the dynamic environment. Additionally, replanning is performed until the goal of the manipulation task is reached. Therefore, the system structure allows the robot to respond even in the case of uncertainty errors in recognition or control.

## Data Availability

The raw data supporting the conclusions of this article will be made available by the authors, without undue reservation.
